# Affective Eye Contact: An Integrative Review

**DOI:** 10.3389/fpsyg.2018.01587

**Published:** 2018-08-28

**Authors:** Jari K. Hietanen

**Affiliations:** Human Information Processing Laboratory, Faculty of Social Sciences, University of Tampere, Tampere, Finland

**Keywords:** affect, arousal, brain, emotion, eye contact, gaze, face, psychophysiology

## Abstract

In recent years, many studies have shown that perceiving other individuals’ direct gaze has robust effects on various attentional and cognitive processes. However, considerably less attention has been devoted to investigating the affective effects triggered by eye contact. This article reviews research concerning the effects of others’ gaze direction on observers’ affective responses. The review focuses on studies in which affective reactions have been investigated in well-controlled laboratory experiments, and in which contextual factors possibly influencing perceivers’ affects have been controlled. Two important themes emerged from this review. First, explicit affective evaluations of seeing another’s direct versus averted gaze have resulted in rather inconsistent findings; some studies report more positive subjective feelings to direct compared to averted gaze, whereas others report the opposite pattern. These contradictory findings may be related, for example, to differences between studies in terms of the capability of direct-gaze stimuli to elicit feelings of self-involvement. Second, studies relying on various implicit measures have reported more consistent results; they indicate that direct gaze increases affective arousal, and more importantly, that eye contact automatically evokes a positively valenced affective reaction. Based on the review, possible psychological mechanisms for the positive affective reactions elicited by eye contact are described.

## Introduction

Other individuals’ gaze is a powerful social stimulus. Gaze direction is used to regulate interaction, to facilitate communicational goals, and to express intimacy and social control, to name some of its important functions in the modulation of social interaction processes (Kleinke, 1986). Most importantly, perhaps, other individuals’ directed gaze signals their direction of attention. We use others’ gaze to discriminate and infer where they have directed their attention—what or who are they looking at. Arguably, the most important discrimination is whether other individuals have directed their eyes toward me or away from me. Seeing other individuals’ eyes directed at me indicates, with a high probability, that they are attending to me, whereas seeing other individuals’ averted gaze signals their attention to be directed away from me.

Extensive lines of research have shown that others’ gaze direction has effects on an observer’s own attention. Direct gaze has been shown to induce attention orienting toward faces (von Grünau and Anston, 1995; Senju et al., 2005; Conty et al., 2006; Doi et al., 2009; Shirama, 2012; Böckler et al., 2014; Lyyra et al., 2017; for a critical view regarding the results from visual search studies, see Cooper et al., 2013), whereas seeing another individual with a gaze directed away from oneself triggers the re-orienting of one’s visuospatial attention in the gazed-at direction (e.g., Friesen and Kingstone, 1998; Driver et al., 1999; Hietanen, 1999; Langton and Bruce, 1999; for a review, see Frischen et al., 2007).

However, the present article aims to review research concerning the effects of others’ gaze direction on observers’ affective responses. This research has received considerably less attention compared to research on the effects of perceived gaze direction on attention. Yet, in our every-day life, we often associate these two. When speaking in front of an audience, a direct gaze cast even by just one of the listeners may feel pleasant and comforting, whereas a gaze aversion by an individual you are approaching in a party makes you feel insecure and uncomfortable. On the other hand, waiting for the last bus at the bus stop at midnight and seeing somebody looking at you may evoke very negative feelings, whereas you feel relaxed when this individual averts gaze away from you. Thus, one’s interpretation of the meaning of another’s gaze is, of course, contingent upon a number of antecedent, concurrent, and anticipated contextual factors. Moreover, the gazer’s verbal and non-verbal behavior, most importantly the verbal content and facial expressions, can have a great influence on the meaning attributed to his or her gaze. In a classic study by Ellsworth and Carlsmith (1968), participants were interviewed by an experimenter, who looked either directly at the participant’s eyes or to her left or right ear a fixed number of times. In addition, the verbal content of the interview was manipulated to be either positive or negative. The results showed that, in the positive context, the participants in the direct gaze group evaluated both the interview and the interviewer more positively as compared to those in the averted gaze group. The result was exactly the opposite in the negative context; the evaluation was more positive in the averted gaze than in the direct gaze group. However, there are many instances and situations that could be regarded as relatively socially neutral. Is there evidence that others’ gaze direction would elicit affective reactions in the observers in these kinds of situations, and if so, is direct gaze (eye contact) perceived as affectively more positive or more negative as compared to averted gaze?

In many animal species, perception of direct gaze triggers protective behavior and elicits threat or fighting responses (Emery, 2000; Skuse, 2003). In humans, direct gaze is used for control, and accordingly, it has been linked to potency, dominance, and power (Argyle et al., 1974; Hall et al., 2005), characteristics that sometimes elicit negative feelings in others. However, humans have a fundamental need for belongingness and for forming and maintaining social relationships, and the fulfillment of these needs is intrinsically positive (Maslow, 1943; Baumeister and Leary, 1995; Eisenberger et al., 2003). Because another’s direct gaze signals attention and social inclusion (Wirth et al., 2010), one could presume that direct gaze would evoke positive affective reactions.

In the present article, I review research in which the effect of another individual’s gaze direction on a perceiver’s affective reactions has been investigated. I will focus on well-controlled laboratory experiments in which other contextual factors possibly influencing perceivers’ affect have been eliminated or minimized. This approach is similar to that employed by numerous studies from the past few decades, in which the recognition of and affective reactions in response to human expressions of emotions—whether expressed in facial expressions, body movements, and posture, or in vocal prosody—were investigated by presenting carefully prepared stimuli to participants. Although the number of studies investigating affective responses to gaze stimuli is still relatively modest, two important themes seem to emerge from this review. The first one is that affective reactions elicited by another individual’s direct versus averted gaze appear to result in different, and often, opposite findings when investigated by using explicit and implicit measures. Second, while explicit evaluations seem to result in heterogeneous findings, studies relying on different types of implicit measures seem to provide a more consistent pattern, indicating that direct gaze evokes a more positive affective reaction as compared to averted gaze.

In the following sections, I will first review the existing research by classifying studies according to the methodology used in measuring participants’ affective reactions. Subsequently, I will deal with different possible explanations for the affective eye contact effect, and finally, I will discuss the possibility that the affective and attentional effects of direct gaze may be intertwined and that the affective reactions elicited by direct gaze should be incorporated into recent models that describe and explain different types of “eye contact” or “watching eyes” effects (cf. Senju and Johnson, 2009; Conty et al., 2016).

## Explicit Affective Feelings Elicited By Gaze Stimuli

Since long, social psychological research has investigated how an individual’s gaze behavior influences other people’s evaluations concerning his or her characteristics. Although these studies did not focus on observers’ affective reactions in response to others’ gaze direction, and although the stimuli often contained many other uncontrolled variables that possibly influenced participants’ evaluations, these studies deserve to be mentioned briefly before focusing on the target studies of the present review. In such studies, participants typically watched films depicting either one individual or two interacting individuals, while the filmed individual’s extent of eye contact with the camera or the other individual appearing on the film was manipulated. In some studies, participants had real, live encounters with collaborators. After watching the films or after the encounters, they were asked to evaluate the target individual on various characteristics. In general, the results showed that an individual making eye contact was evaluated more favorably as compared to an individual not making eye contact. Additionally, within limits, the degree of positive evaluations correlated positively with the extent of eye contact. These evaluations included characteristics such as likability, competence, attractiveness, intelligence, credibility, and potency (e.g., Argyle et al., 1974; Abele, 1981; Shrout and Fiske, 1981; for reviews, see Kleinke, 1986; Hall et al., 2005). Within this research tradition, other studies presented well-controlled facial stimuli with neutral expressions to participants and the researchers manipulated only the gaze direction. The results from these types of studies revealed an association between direct gaze and more positive evaluations. For example, higher liking ratings were observed for faces with direct versus averted gaze, both when using photographs of real people (Mason et al., 2005) and virtual avatars (Kuzmanovic et al., 2009) with dynamic gaze shifts (e.g., gaze shifting from averted to direct gaze or vice versa).

Importantly for the present review, there are also few studies in which participants were asked to directly evaluate their own affective feelings in response to neutral faces with direct and averted gaze. However, unlike the studies described above, the results from these studies seem to reveal a much more inconsistent pattern of the effects of gaze direction on affective responses. Wirth et al. (2010) showed participants 2.5-min “movies” with direct and averted gaze faces. In the direct-gaze stimuli, photographs of real faces with direct gaze were interspersed with occasional closed eyes pictures (i.e., creating an illusion of blinking eyes), whereas, in the averted-gaze stimuli, the gaze was alternating between the stimulus face looking to the left and right. After watching the movies, participants were asked to rate, among other things, their positive mood (friendly, happy, and good) and negative mood (unpleasant, sad, bad, and unfriendly). The results showed that the participants who watched the averted gaze film (a between-subject design) reported significantly more negative feelings than did those who watched the direct gaze film. Further, the gaze direction did not influence participants’ ratings of their positive feelings. More recently, feelings of distress and of being excluded were measured in a study in which participants were presented photographs of faces with direct or averted gaze (Leng et al., 2018). A single trial consisted of a stimulus sequence of direct gaze (1000 ms) and closed-eyes (800–1000 ms) followed by either direct gaze or averted gaze (left or right, all presented for 1500 ms). All participants were presented with both types of sequences (a within-subject design). The results showed that participants felt more distressed and more excluded when looking at the sequences ending with averted gaze as compared to sequences ending with direct gaze. However, it should be noted that, in these studies, the affective feelings were rated after participants had been asked to evaluate the extent to which they were looked at by the stimulus face (Wirth et al., 2010) and the extent to which they felt ignored and excluded while watching the stimuli (feelings of ostracism). It is possible that these rating tasks influenced participants’ responses regarding their affective responses.

Despite these possible confounding factors, however, compatible results have been observed in studies without preceding tasks that may have led to biased affective ratings. Faces with static direct gaze have been shown to elicit more pleasant subjective feeling states (i.e., higher ratings of subjective affective valence) as compared to faces with averted gaze, both when pictures of real human faces (Uono and Hietanen, 2015) or pictures showing the eye-region of animated realistic looking faces (Experiment 2, Chen et al., 2017a) were used as stimuli. However, it should be noted that the study by Chen and colleagues also included eye-region stimuli with closed eyes. In fact, these closed-eyes stimuli elicited even higher valence ratings than direct-gaze stimuli did. In a study by Marschner et al. (2015), virtual characters shifted their gaze (dynamic gaze shifts) ending up with either a direct or an averted gaze. In this study, the facial expression was also manipulated. The results showed that, when embedded in a neutral facial expression, gaze direction did not have an effect on participant’s ratings of their subjective affective valence. However, in the context of happy faces, direct gaze increased valence ratings; whereas, in the context of angry faces, direct gaze decreased valence ratings in comparison to averted gaze (Marschner et al., 2015).

In two studies, Hietanen and colleagues presented neutral faces with direct and averted (static) gaze in two different presentation modes; either live (presented through a liquid crystal window) or as images on a computer monitor, and they compared the effects of gaze direction on self-ratings of affective valence between these modes of stimulus presentation. In both studies, the gaze direction in the images did not have any effect on subjective valence ratings; whereas, for live faces, direct gaze elicited lower affective valence (albeit still positive) as compared to averted gaze or closed eyes (Hietanen et al., 2008; Pönkänen et al., 2011a).

To sum up, at this stage of research, it is difficult to analyze the reasons for the diverse effects observed in different studies. So far, only a few studies have been conducted on this topic, with several differences in the stimuli used. Interestingly, however, most of these studies relied on the same method to measure subjective affective feelings; the Self-Assessment Manikin (SAM) scales for affective valence (unpleasant–pleasant) and affective arousal (calm–aroused) (Bradley and Lang, 1994). On attempting to identify a pattern behind the studies and their results described above, one would be tempted to argue that the more the stimuli resembled the gaze during natural interaction, the less likely direct gaze was to evoke relatively more positive affective feelings as compared to averted gaze. Studies in which participants were presented still images of faces or eye-regions (Uono and Hietanen, 2015; Chen et al., 2017a) reported higher valence ratings to direct versus averted gaze. A study that presented stimuli with dynamic gaze aversions resulted in no effect of gaze direction (Marschner et al., 2015). Studies using real, live faces as stimuli resulted in lower valence ratings to direct than averted gaze did (Hietanen et al., 2008; Pönkänen et al., 2011a). In one of these previous studies, it was suggested that lower valence ratings to direct than averted gaze when facing another, live individual could be due to the enhanced feelings of self-involvement and of the uneasiness caused by being watched by another individual (Pönkänen et al., 2011a). Thus, one could speculate that the more capable the stimuli are of evoking the feeling of being looked at by another, the less positive are the conscious self-evaluations regarding the valence of one’s affective feeling state. However, of course, this is a very speculative suggestion considering the present stage of research.

In the following sections, I review research that has employed different types of implicit measures to investigate affect-related responses to another individual’s direct and averted gaze. These measures include both behavioral and physiological measurements. As we will see, these studies seem to provide a more consistent pattern of findings regarding the effects of gaze direction on affective reactions.

## Behavioral Paradigms with Implicit Measures

Lawson (2015) conducted seven experiments by using the Implicit Association Test (IAT) to measure implicit affective evaluations of direct and averted gaze. In the IAT, participants’ task is to categorize stimuli belonging to two pairs of categories. Lawson asked participants to categorize pictures of faces in which the individual was looking either toward or away from them (static gaze), and to classify affectively positive and affectively negative words. Two response keys were used. In one condition (“congruent” sorting condition), the task instructions required participants to press one key if the stimulus was a face looking toward or if the stimulus was a positive word, and to press the other key if the stimulus was a face looking away or if the stimulus was a negative word. In the other condition (“incongruent” sorting condition), the associations between the response key nominations and the categories were changed, i.e., looking toward/negative word vs. looking away/positive word. By comparing the speed of categorization in the two sorting conditions, it is possible to investigate the strength of implicit associations between the target categories (i.e., direct and averted gaze) and the positive and negative valence of the words (Greenwald et al., 2003; Nosek et al., 2005). The results showed that the categorization times were shorter in the congruent than in the incongruent conditions. In other words, participants more easily implicitly associated faces looking toward (direct gaze) and faces looking away (averted gaze) with positivity and negativity, respectively, than the other way around. Importantly, this pattern of results was observed when the faces were shown in full frontal view, when the head was rotated to the left or right side, and even when the frontal-view faces were presented upside-down. Strikingly, even angry faces with direct and averted gaze were more readily associated with positivity and negativity, respectively, than the other way around. These results are in striking contrast with those of studies relying on self-reports. As cited above, a study by Marschner et al. (2015) showed that, while direct gaze increased valence ratings for happy faces in comparison to averted gaze (but no effect of gaze direction on neutral faces), direct gaze decreased valence ratings for angry faces. Lawson (2015) also tested whether similar results would be obtained if the face stimuli were replaced by arbitrary geometrical shapes of two different colors (associated with “looking at you” and “looking to the side” labels) or by arrows pointing toward or away. Even in these conditions, participants implicitly evaluated “looking at you” stimuli more positively than “looking to the side” stimuli, albeit the magnitude of this effect was significantly smaller than that observed in the experiments that used facial stimuli.

In the IAT study by Lawson (2015), the task instructions directed participants’ attention to the gaze direction, and the task instructions activated the concepts of “looking at you” and “looking to the side” because the face stimuli were presented with these labels and the response selection was based on discriminating between these categories. Thus, an important question is whether faces with direct gaze, compared to faces with other gaze directions, would also be more positively associated in conditions in which the task instructions do not require participants to attend to the gaze direction and do not imply that the stimuli are related to “looking at you” and “looking to the side.”

Dubey et al. (2015) used a novel choose-a-movie (CAM) paradigm in which participants saw two colored boxes on the screen in each trial. They could open one of the boxes (with a varying number of locks) and then watch a movie clip associated with that box. During preceding familiarization trials, participants had learnt the mapping between the color of the box and the category of the movie that was shown when the box was opened. Three categories of movies were prepared. In direct gaze movies, an individual looked up (toward the camera) and smiled. Averted gaze movies showed exactly the same stimulus, but they were filmed with a camera positioned such that the individual appeared to be looking away from the camera. In object movies, household objects were slowly rotating on a turntable. The results showed that participants were prepared to put in more effort to watch direct gaze movies as compared to averted gaze (and object) movies. The authors interpreted their results in the context of the social motivation and reward gained from seeing social stimuli, but the results can also be interpreted to reflect more positive affective reactions to direct versus averted gaze. Interestingly, Dubey et al. (2015) also examined the same in individuals with autism spectrum disorder (ASD), and these adults showed a significant reduction in their preference for direct gaze.

Chen et al. (2017a) employed the affective priming paradigm to investigate the automatic affective evaluations elicited by gaze stimuli. In this study, direct-gaze, averted-gaze, and closed-eyes stimuli (eye-region “letterbox” stimuli) were briefly presented (masked 13-ms and unmasked 100-ms presentation times) as primes, followed by positive and negative words as targets (with prime-target onset delays of 150 ms and 300 ms). Participants’ task was to ignore the primes and to evaluate the words as affectively positive or negative, as quickly as possible. Thus, unlike in the study by Lawson (2015), in this study, participants’ attention was not directed to the gaze direction, and concepts related to the stimulus face’s focus of attention were not activated. The results by Chen et al. (2017a) showed that the response (categorization) times for positive words were significantly shorter when they were presented after direct-gaze rather than closed-eyes primes, whereas the response times to negative words were significantly shorter when they were presented after closed-eyes rather than direct-gaze primes. For both targets, the response times after averted-gaze primes were numerically between those observed after direct-gaze and closed-eyes primes, but these response times did not differ statistically significantly from those.

In the affective priming literature, the effect of prime category on the affective categorization of targets is typically interpreted to show that the prime automatically activates affective evaluation and facilitates the processing of affectively congruent targets (Fazio, 2001; Klauer and Musch, 2003). Thus, Chen et al. (2017a) interpreted their results to indicate that seeing a direct gaze automatically activated more positive evaluations than did seeing closed eyes. Interestingly, Chen and colleagues also measured explicit affective feelings evoked by the stimuli. In Experiment 1, after the affective priming experiment, participants rated the affective valence of their subjective feelings in response to the prime stimuli. Intriguingly, these results showed exactly the opposite pattern of results; more positive evaluations to closed-eyes than to direct-gaze stimuli. Experiment 2 confirmed this pattern of results for the explicit ratings independent of whether the stimuli were shown briefly (100 ms, similar to when shown as primes in the affective priming paradigm) or for a longer time (until an explicit rating response was made). The authors concluded that their results indicated that the perception of mere eye gaze automatically activates observers’ emotions and that the instinctual “gut feeling” to eye contact is positive.

The three studies described in this section provide consistent support for a view that direct gaze is implicitly associated with a more positive affect and that it automatically activates a more positive affective reaction as compared to averted gaze. The study by Lawson (2015) showed this even when the direct and averted gazes were embedded in an angry face. This finding would suggest that another individual’s self-directed attention, even if associated with a hostile intention, would be more positively evaluated as compared to not receiving his or her attention. However, it is possible that due to the behavioral task, the participants’ attention was focused on gaze direction to such an extent that the effect of the facial expression was minimized. Moreover, the effect of facial expression might have been minimized because the participants were presented faces with only one type of expression during the experiment, either angry faces or happy faces (between-subject design). In future, it would be advisable to investigate if facial expressions modulate the effect of gaze direction on affective reactions when participants are presented expressions from more than just one category of emotions (in a within-subject design), and when the task instructions do not draw participants’ attention to the gaze direction. This could be achieved by employing the affective priming paradigm, for example.

## Autonomic Arousal and Amygdala Activation

Physiological arousal is a fundamental component of affective responses (Plutchik, 1980). Several studies have reported that sympathetic skin conductance responses (SCRs)—a robust indicator of affective arousal (Critchley, 2002)—are greater in conditions with another individual’s direct gaze rather than averted gaze or closed eyes (e.g., Nichols and Champness, 1971; Hietanen et al., 2008; Helminen et al., 2011; Pönkänen et al., 2011b; Myllyneva and Hietanen, 2015). Pupil dilation, another index of physiological arousal, has also been shown to be larger in response to direct- versus averted-gaze stimuli (Porter et al., 2006). Further, in another study, the dynamics of the pupil dilation response correlated with the length of time participants felt comfortable to look at faces with direct gaze; the longer periods of direct gaze participants preferred, the faster was the increase in pupil dilation (Binetti et al., 2016).

Affective arousal is controlled by the amygdala (Mangina and Beuzeron-Mangina, 1996; LeDoux, 2000; Williams et al., 2005; Laine et al., 2009). Thus, in line with the psychophysiological findings mentioned above, amygdala activation has been linked with the processing of gaze direction as well as emotion. Imaging studies in humans have shown that, not only are amygdala responses to facial emotional expressions modulated by gaze direction (Adams et al., 2003; Sato et al., 2004; Hadjikhani et al., 2008; Ewbank et al., 2010; Adams et al., 2012), but amygdala activation is also responsive to gaze direction in emotionally neutral faces. Studies have reported greater right amygdala activation in response to direct rather than averted gaze (Kawashima et al., 1999; Wicker et al., 2003). Recently, a study examined amygdala activation in healthy participants and in a cortically blind patient, and the results showed greater activation in the right amygdala in response to images of (neutral) faces with direct gaze as compared to faces with averted gaze, both in healthy participants and in the cortically blind patient (Burra et al., 2013). These findings suggest that amygdala responsivity does not even require an intact primary visual cortex. Other studies have also shown that functional coupling between activations in the right fusiform gyrus, an area specialized in face processing, and the right amygdala is greater for direct than for averted gaze (George et al., 2001).

However, even if increased physiological arousal and amygdala activation to direct gaze is interpreted to reflect an affective response, it is more difficult to say anything about whether this response is related to a positive or negative affective response. Unlike earlier views that associate the amygdala with the processing of negative (threatening) information, more recent views have emphasized its role in processes related to affective arousal and affective attention, both positive and negative (Hoffman et al., 2007; Pessoa, 2010). For example, intra-cerebral event-related potentials recorded from the human amygdala have shown enhanced responses to eye-region stimuli expressing both fear and happiness (Meletti et al., 2012). Moreover, other studies have shown the opposite pattern of results; greater amygdalar activation in response to averted than to direct gaze (e.g., Straube et al., 2010; Sauer et al., 2014). Thus, greater amygdala activation in response to direct versus averted gaze is difficult to interpret in terms of valenced affective reactions.

Nevertheless, it is evident that the amygdala plays a central role in mediating the affective arousal response and attentional allocation to direct gaze. A subcortical processing tract from the superior colliculus to the amygdala, through the pulvinar, is likely to be involved in detecting eyes and processing information about gaze direction (Senju and Johnson, 2009; Tamietto et al., 2012; Nguyen et al., 2014, 2017; Soares et al., 2017). In a functional magnetic resonance imaging (fMRI) study conducted on rhesus monkeys, Hoffman and colleagues showed that a part of the amygdala, called the lateral extended amygdala (LEA, comprising the central nucleus and the bed nucleus of the stria terminalis) was specifically sensitive to gaze direction (Hoffman et al., 2007). The central nucleus of the amygdala sends fibers to centers controlling autonomic arousal (LeDoux, 2000; Laine et al., 2009), and therefore, it is thought to play a central role in heightening arousal and orienting attention (Davis and Whalen, 2001). Interestingly, in Hoffman et al.’s (2007) study on monkeys, LEA activation was stronger in response to averted rather than direct gaze, and recordings of SCRs also showed greater responses to averted rather than direct gaze. However, as cited above, several studies in humans have reported greater SCRs in response to seeing another individual’s direct gaze rather than averted gaze. Based on these results, it could be presumed that, in humans, the LEA plays a central role in increased autonomic arousal responses and attention orienting to direct gaze. Dysfunction in these nuclei could result in direct gaze not being affectively arousing and not grabbing visual attention. Studies with patients suffering from amygdala lesions have shown that they do not look at the eye region the same way as controls do (Spezio et al., 2007), and that they do not show gaze-cued attention orienting (Akiyama et al., 2007).

## The Brain Reward Network

Some neuroimaging studies have reported the effects of gaze direction on the activation of the brain systems implicated in the processing of reward. The “classic” reward network includes the ventral striatum (nucleus accumbens and ventral pallidum) and the orbitofrontal cortex, but areas such as the insula and anterior cingulate cortices have also been suggested to be parts of this network (Rolls, 2000; Schultz, 2006; Berridge and Kringelbach, 2015). Now, as another individual’s direct gaze signals his or her communicative intent and social inclusion, and as imaging studies have shown that social interaction with others activates the striatum (Báez-Mendoza and Schultz, 2013; Pfeiffer et al., 2014), one would expect that seeing another individual’s direct gaze would activate the reward system.

In an event-related fMRI study by Kampe et al. (2001), participants were shown images of faces with their eyes directed either at or away from them. After the imaging session, participants were instructed to rate the attractiveness of the stimulus faces. The results showed that, indeed, the gaze direction had an effect on the activation of the ventral striatum. However, interestingly, this activation was also dependent on facial attractiveness. For stimuli with direct gaze, ventral striatum activation increased as a function of facial attractiveness, whereas, for averted-gaze stimuli, activation decreased with increasing attractiveness (Kampe et al., 2001). Therefore, the authors suggested that facial attractiveness acted as a social reward. A direct gaze from an attractive face signals a possibility for an upcoming social interaction with an attractive individual, and thus, it anticipates a social reward. Instead, a direct gaze from an unattractive face may lead to the anticipation of an unwanted social interaction. Thus, so far, direct gaze has not been shown to activate the ventral striatum. Therefore, future studies need to examine if the activation of the ventral striatum in response to the direct gaze of faces occurs irrespective of their attractiveness. Further, in Kampe et al.’s (2001) study, the face stimuli were images of static faces. Therefore, it is possible that, for example, dynamic shifts of gaze toward the viewer could elicit enhanced ventral striatum activation independent of facial attractiveness.

In fact, in one study, anterior insula activation was observed only in response to dynamic shifts of gaze, but not in response to static images (Ethofer et al., 2011). Ethofer et al. (2011) measured participants’ brain activation in response to dynamic gaze when they were performing a gender categorization task. Findings revealed that gaze shifts toward the viewer resulted in greater activation within the right anterior insula as compared to gaze shifts away from the viewer. Interestingly, a connectivity analysis revealed an increase in the functional coupling of the right posterior superior temporal sulcus (pSTS)—a central region in gaze processing—with the anterior insula when the gaze shifted toward rather than away from the viewer. Notably, there was also a highly significant difference between the hemispheres in terms of the structural connectivity between the pSTS and the anterior insula. Specifically, in the left hemisphere, only infrequent connections were found between the pSTS and anterior insula. Further, in a study in which the participants were looking, via a large mirror, at a live individual sitting in the scanning room, greater activation in the anterior insula, anterior cingulate, and globus pallidus was reported in response to direct rather than averted gaze (Cavallo et al., 2015). Finally, in a study measuring electroencephalographic activity in response to dynamic gaze shifts, source localizing analyses showed a cluster of sources in the orbitofrontal cortex, in which the activity was greater in response to the dynamic gaze that shifted from averted to direct gaze than from direct to averted gaze, specifically between 190 and 220 ms after stimulus onset (Conty et al., 2007).

In sum, neuroimaging studies have shown that seeing a direct gaze results in greater activation of the various components of the reward system as compared to seeing an averted gaze. These results could be considered as evidence supporting the view that gaze direction can trigger affective processing and that direct gaze elicits more positive affective reactions compared to those elicited by averted gaze. However, great cautiousness is warranted in interpreting these results. Apart from the ventral striatum, the association between reward and the functioning of the other brain areas mentioned above is complicated by the fact that these areas are also involved in many other cognitive, affective, and interoceptive functions, and, at the present stage of research, it is difficult to know whether the findings described above are related to affective reactions elicited by gaze or to some other processes like self-referential processing (see, e.g., Northoff et al., 2006; Herbert et al., 2011).

## Asymmetric Frontal Cortical Activity

More direct brain research evidence associating gaze direction with affective valence comes from studies reporting the effects of gaze direction on the activation of the brain systems implicated in the processing of affect and motivational states. There is a considerable line of research associating asymmetric frontal alpha-band electroencephalographic (EEG) activity to emotional and motivational processes. The relatively greater activation of the left versus the right frontal cortex has been linked to positively valenced affect and activation of the approach-related motivational system, whereas the opposite pattern of frontal asymmetric activation has been linked to negative affect and activation of the avoidance system (Davidson, 1984, 2004; Harmon-Jones, 2003, 2004; Harmon-Jones et al., 2006; Van Honk and Schutter, 2006). Most of this research has investigated the association between resting state frontal EEG activity, and trait affect and trait motivation, but other studies have examined asymmetric frontal EEG activity in response to affective and motivationally significant stimuli (for a review, see Harmon-Jones and Gable, 2018).

Few studies have shown that seeing another individual’s gaze direction has an effect on observers’ frontal EEG asymmetry. For instance, Hietanen and colleagues measured the hemispheric asymmetry in the frontal EEG activity in response to seeing another, live individual with direct and averted gaze (Hietanen et al., 2008; Pönkänen et al., 2011b). Findings revealed that seeing another individual’s direct gaze elicited greater relative left-sided frontal EEG activity as compared to seeing averted gaze. These results provide evidence in favor of the fact that direct gaze elicits greater activation in brain mechanisms associated with approach motivation and positive affect as compared to averted gaze. In fact, in Hietanen et al.’s (2008) study, another individual’s averted gaze elicited right-sided, avoidance-related frontal EEG asymmetry. Interestingly, in their study, subjective ratings of affective valence were also measured, and they indicated that averted gaze was rated as slightly more pleasant as compared to direct gaze. Thus, in this study too, implicit (physiology) and explicit (self-rating) measures resulted in incongruent patterns of results.

The frontal EEG asymmetry response to gaze direction has been shown to be modulated by personality and neuro-psychiatric disorders. Uusberg et al. (2015) measured EEG asymmetry in response to a live individual’s gaze in participants with varying degrees of neuroticism according to the Five Factor Model. The results showed that, in participants scoring low on neuroticism, direct gaze elicited greater left-sided frontal EEG asymmetry as compared to averted gaze, as observed in the two studies mentioned above. However, in participants scoring high on neuroticism, direct gaze elicited greater right-sided frontal EEG asymmetry as compared to averted gaze. In another study, the frontal EEG asymmetry response to gaze direction was investigated in adolescents with clinically diagnosed social anxiety disorder (Myllyneva et al., 2015). The results showed marginally greater left-sided frontal EEG response to direct gaze in control participants as compared to the clinical group. ASD have also been shown to influence the frontal EEG asymmetry response to gaze. Kylliäinen et al. (2012) investigated children with ASD and control children, and showed that, in the control children, direct gaze elicited greater left-sided frontal asymmetry than closed eyes did; whereas, in ASD children, the gaze direction did not have an effect on frontal EEG asymmetry responses. These three studies indicate that the increased negativity to direct gaze associated with neuroticism, social anxiety, and autism (Campbell and Rushton, 1978; Senju and Johnson, 2009; Moukheiber et al., 2010) is reflected in the frontal EEG asymmetry responses.

## Startle Reflex Modulation

The startle reflex is an automatic defensive reaction to abrupt and strong stimuli. A convenient way to investigate the startle reflex is to measure electromyographic (EMG) eyeblink responses (Lang et al., 1990; Bradley et al., 1999; Grillon and Baas, 2003) or heart rate (HR) acceleration responses (Graham and Clifton, 1966; Graham, 1992; Holand et al., 1999; Richter et al., 2011) triggered by an acoustic startle probe. Interestingly, simultaneously presented affective foreground stimuli can modulate the magnitude of the reflex. The eyeblink and the cardiac acceleration responses are increased in an unpleasant context and decreased in a pleasant context (e.g., Vrana et al., 1988; Bradley et al., 1993; Bradley and Lang, 2000; Ruiz-Padial et al., 2005; Roy et al., 2009; Sánchez et al., 2009; Ramírez et al., 2010; Richter et al., 2011).

Two studies have investigated the modulatory effect of perceived gaze direction on the magnitude of the startle reflex. In one study, acoustic startle probes were presented to male participants while pictures of nude females with direct and averted gaze were shown as foreground stimuli (Lass-Hennemann et al., 2009). Affectively positive nude bodies decreased participants’ eyeblink response. The gaze direction also had an effect, in that the attenuation was smaller for pictures with direct rather than averted gaze. This would suggest that averted gaze was perceived as more positive than direct gaze was. However, the authors suggested that the effect of gaze direction was due to its effect on attention. Direct gaze grabbed attention to the faces and therefore, the effect of the nude bodies was reduced in the context of direct gaze.

More recently, the effect of gaze direction on startle reflex modulation was investigated by presenting loud auditory stimuli while a live model’s direct- and downward-gaze stimuli were presented through a liquid crystal window (Chen et al., 2017b). In this study, both eyeblink startle and cardiac reflexes were measured. The results showed that the magnitude of the eyeblink startle and cardiac reflexes decreased when measured in the context of a direct versus downward gaze. Interestingly, in this study, the participants also self-evaluated the valence of their subjective feelings while looking at the stimulus faces. Similar to other previous studies measuring both explicit and implicit affective reactions (e.g., Hietanen et al., 2008; Chen et al., 2017a), this study found that direct gaze was rated as slightly less positive as compared to downward gaze, although the difference was not statistically significant. In sum, the results of this study provide further evidence in support of the view that another individual’s gaze direction elicits affective reactions, and that, compared to averted gaze, direct gaze automatically elicits more positive affective responses in the viewer.

## Facial Electromyography

Measurements of EMG responses from the facial muscles involved in producing facial emotional expressions have been widely used as a method to investigate the valence of automatic affective reactions (Cacioppo et al., 1986; Tassinary and Cacioppo, 1992; Dimberg and Thunberg, 1998; Dimberg et al., 2000). Affectively positive stimuli increase the activity of the Zygomaticus major (smile) and decrease activity of the Corrugator supercilii muscle (furrows between the eyebrows), whereas negative stimuli increase the activity of the Corrugator supercilii muscle (Cacioppo et al., 1986; Larsen et al., 2003).

Previous studies have reported that an expressor’s gaze direction can modulate the facial EMG responses elicited by the emotional facial expression (Schrammel et al., 2009; Rychlowska et al., 2012; Soussignan et al., 2013), but, in these studies, no effect of gaze direction was observed in response to neutral faces. Hietanen et al. (2018) argued that the reason for the lack of a mere gaze direction effect could be that, in these previous studies, the stimuli were images of human faces or animated virtual characters. A viewer knows that an image of a face presented on a computer monitor does not look back. This argument was supported by their previous experiments that showed that, while psychophysiological responses (electroencephalographic and autonomic responses) to direct versus averted gaze had been observed to differ when a live individual was presented as a stimulus, there was no effect of gaze direction on responses to pictures of the same individual (Hietanen et al., 2008; Pönkänen et al., 2011a,b). Therefore, Hietanen et al. (2018) investigated the effect of another individual’s gaze direction on participants’ facial responses by showing a live individual with a neutral expression as a stimulus. In their study, not only did the model individuals vary their gaze direction, but the participants were also allowed to look either directly at the model individual or slightly away from him or her, at a pre-determined fixation spot. This lateral fixation spot was placed such that the participants were able to see, from the corner of their eye, whether the model individual had a direct gaze or not. The results showed that the zygomatic responses were greater in response to another individual’s direct versus averted gaze when the participant was looking toward the other as well as when the participant was looking slightly away. However, the participant’s own gaze direction also had an effect; the zygomatic response to the model’s direct gaze was greater during the former (i.e., a genuine eye contact) as compared to the latter condition.

Thus, measurements of facial EMG responses have also provided evidence that, in a neutral context, another individual’s gaze direction elicits affective reactions, and that, compatible with the other findings reviewed above, direct gaze seems to elicit a positive affective reaction. However, as Hietanen et al. (2018) stressed in their discussion, we cannot know for sure about the extent to which the observed facial reactions reflect automatic affective reactions or highly automatized affiliative facial responses triggered by communicative motivations during social interaction.

## The Affective Eye Contact Effect: Possible Mechanisms

The present review has shown that studies using explicit and implicit measures have provided somewhat contradicting findings regarding whether direct gaze elicits more positive or less positive affective reactions as compared to control-gaze stimuli. Many of the studies relying on explicit self-evaluations reported higher valence ratings to averted-gaze or closed-eyes stimuli as compared to ratings in response to direct-gaze stimuli, whereas studies using different kinds of implicit measures consistently showed more positive affective reactions to direct gaze than to averted gaze. How can we explain these discrepancies in the results of explicit and implicit measurements?

People’s explicit responses are known to be susceptible to motivational biases and individuals may lack introspective access to their implicit affective reactions. In fact, correlations between explicit and implicit measures increase as a function of increasing spontaneity of self-reports (Hofmann et al., 2005). There is plenty of evidence in the area of social cognition research on how explicit and implicit processes can be not only complementary but also oppositional (Frith and Frith, 2008). Introspection of one’s own feelings to direct-gaze stimuli may, for example, evoke uncertainness because one cannot be sure about the gazer’s intentions and the reasons for being the target of his or her attention. Another individual’s direct gaze may also increase self-directed attention and self-awareness (Hietanen and Hietanen, 2017). This, in turn, may lead to critical evaluation of the self and to a negative affective state (Duval and Wicklund, 1972). Thus, even if one’s initial and automatic response to direct gaze was affectively positive, it could be suppressed by more controlled evaluations, and it may even be biased in a negative direction. This idea is compatible with the views proposing that socio-cognitive functions depend on the workings of two systems; one responsible for the detection of socially relevant actions, which relies on automatic processing; and another responsible for social evaluation, which relies on more controlled processing (Spunt and Lieberman, 2013; Vogeley, 2017).

**FIGURE 1:**
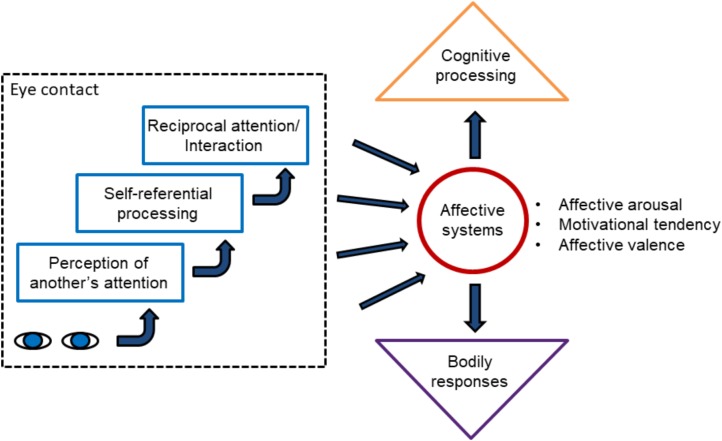
The figure summarizes different possible factors behind the affective eye contact effect. Detection of a pair of eyes directed to the self initiates a gaze shift toward the eyes, thus leading to eye contact. It could also trigger affective processing mediated by subcortical (the superior colliculus, pulvinar, and amygdala) and cortical visual systems. Eye contact also triggers mentalizing processes in the observer involving a belief that the self is attended by the other. This belief leads to enhanced self-referential processes. An understanding that the other individual perceives to be attended by the self (i.e., by the observer) leads to reciprocal attention and interaction. All these processes may contribute to the activation of affective systems. Activation of the affective systems influences cortical cognitive processing, resulting in, for example, the affective priming effects. Further, it initiates affective bodily responses via the amygdala and other subcortical centers, for example, affective arousal. The figure illustrates hypothetical mechanisms of automatic affective reactions in response to eye contact. As suggested in the main text, automatic affective reactions can be suppressed when one explicitly evaluates his or her affective feelings during eye contact.

Thus, the reviewed research provides considerably strong evidence that eye contact automatically elicits positive affective reactions. However, an essential question that emerges is *why* eye contact triggers positive affective reactions. In the following paragraphs, four different possible factors behind the affective eye contact effect are characterized. For an illustration, see **Figure 1**.

In principle, affective reactions to gaze could be triggered by the perception of eyes—by low-level visual cues related, e.g., to luminance distribution (Kobayashi and Kohshima, 1997; Langton et al., 2000; Ando, 2002) analyzed from the eyes—processed by subcortical mechanisms described in the section dealing with amygdala activation in response to gaze. According to the fast-track modulator model of eye contact presented by Senju and Johnson (2009), direct gaze is detected and processed by a subcortical pathway, involving the superior colliculus, pulvinar, and amygdala. Senju and Johnson (2009) suggested that this pathway then modulates the functioning of the social brain network and the network involved in mentalizing. In the context of the present review, one could argue that the subcortical pathway also modulates the networks involved in regulating affective responses. Thus, according to this view, the affective reactions in response to gaze would be triggered by the visual information analyzed from the gazers’ eyes.

In the introduction of this article, when discussing about why gaze direction should be expected to have any affective effects, a higher-level explanation was suggested based on human beings’ fundamental need for belongingness and for forming and maintaining social relationships (Maslow, 1943; Baumeister and Leary, 1995; Eisenberger et al., 2003). It was suggested that, as direct gaze indicates attention and social inclusion (Wirth et al., 2010), it is likely to be perceived as a positive social signal. Thus, another possibility is that, rather than based on visual information from the eyes, the affective effects reflect the “perception” of other individuals’ attention directed to the self. In fact, there is evidence supporting this latter view. First, previous studies have shown greater autonomic arousal responses and greater relative left-sided frontal EEG activity in response to direct versus averted gaze when participants saw a live individual, but not when they saw an image of a face on a computer monitor (Hietanen et al., 2008; Pönkänen et al., 2011b). As noted above, images do not look back. More direct evidence in support of this view was provided by Myllyneva and Hietanen (2015, Experiment 1), who measured SCRs in response to a live individual’s gaze direction in two conditions. In one condition, the participant and the model individual were able to see each other normally; whereas, in the other condition, the participant was led to believe that a half-silvered mirror was placed between the participant and the model in such a way that the model could not see the participant. The results showed greater SCRs to direct than to averted gaze when the participants believed that the model was able to see them, but not when the participants believed that the model could not see them. Moreover, in Experiment 2, the authors manipulated the visibility of the model’s eyes. In three different experimental blocks, the model wore a different pair of sunglasses: a pair without lenses (eyes visible), a pair of normal sunglasses with dark lenses (eyes not visible, but the participants knew that the model was able to see them), and an identical pair of sunglasses with dark lenses, but with lenses covered from inside (eyes not visible and the participants knew that the model was not able to see them). The results showed greater SCRs to direct gaze/head orientation as compared to averted gaze/head orientation, both when the eyes were visible and when the participants were wearing normal sunglasses. However, when the model was wearing opaque sunglasses, there was no effect of gaze direction/head orientation on SCRs. These results strongly indicate that the enhanced physiological responses to another individual’s direct gaze reflect the awareness of being attended to by another individual, rather than as responses to the visual appearance of directly looking eyes.

A third possible mechanism relates to the enhanced self-directed attention triggered by eye contact. Recently, Conty et al. (2016) postulated that eye contact initiates, via self-directed attention, a self-referential mode of information processing, i.e., a heightened processing of stimuli in relation with the self. This postulation was confirmed later, based on the findings of a study that showed that, in a task requiring participants to complete sentences by choosing a pronoun (first singular, first plural, third singular, or third plural), a gaze stimulus presented before each trial influenced the selection of the pronouns; specifically, direct gaze (eye contact) increased the use of first-person pronouns (Hietanen and Hietanen, 2017). Now, as self-referential processing is associated with positive affect (self-positivity) (Baumeister, 1998; Heine et al., 1999), it could be postulated that the positive affective reactions elicited by eye contact could be mediated by the effects of eye contact on self-reference.

A fourth possibility is that the affective responses to gaze reflect responses to interaction. Recently, in the field of social attention, particularly in studies investigating eye movements and fixations when looking at real people versus images, it has been reported that the gazing patterns can be very different between these conditions. It has been suggested that the key difference between watching pictorial and live stimuli is in the possibility for bidirectional sending and receiving information, i.e., possibility for interaction (Laidlaw et al., 2011; Risko et al., 2012; Wu et al., 2013). Possibility for interaction has also been suggested to play a role in triggering the autonomic affective responses to eye contact (Myllyneva and Hietanen, 2016). In a recent experiment, as described in the previous section, Hietanen et al. (2018) measured participants’ facial EMG responses when they were allowed to look either directly at the model individual or slightly away from him or her. As described above, the zygomatic response to the model individual’s direct gaze was greater when participants were looking at the model as compared to when their gaze was slightly averted. Thus, despite the fact that, in both conditions, participants were able to see that the model’s attention was on them (belief of being seen), the reciprocated direct gaze (eye contact) resulted in the strongest zygomatic response. Moreover, in that study, Hietanen and colleagues also measured SCRs, which revealed that the autonomic arousal response was greater to the model’s direct versus averted gaze only when participants were looking toward the model individual, but not when they were not reciprocating the direct gaze. The authors interpreted this finding to suggest that enhanced affective arousal to another individual’s direct gaze is conditional to (a) an observer’s perception and understanding that another individual’s attention is directed to him or her and (b) an observer simultaneously directing his or her own gaze toward the other individual and understanding that the latter perceives that he or she is being seen by the observer.

Thus, it is possible that, for example, the previous results by Myllyneva and Hietanen (2015) showing no effect of another individual’s gaze direction (direct versus averted gaze) on affective arousal responses when the participant believed that the model could not see him or her, was not only due to the self’s understanding of not being seen by the other, but also due to self’s understanding of not being able to communicate to the other that “I am looking at you.” Even though looking at each other’s eyes between two motionless individuals does not involve “behavior” as such, there is nevertheless an interaction—coordinated, reciprocal, and joint activity (for theoretical definition of interaction, see De Jaegher et al., 2010). In eye contact, the parties have chosen to simultaneously direct their attention toward the other and they both know about it. De Jaegher et al. (2010) emphasized that an essential characteristic of interaction is the engagement between the agents. Genuine eye contact definitely fulfills this criterion. Schilbach and colleagues have also emphasized the importance of interaction and emotional engagement as a fundamental factor differentiating between natural encounters with another individual and situations where an individual is merely observing another without a possibility to interact (Gangopadhyay and Schilbach, 2012; Schilbach et al., 2013).

At the present stage of research, it is difficult to evaluate the relative importance of the possible mechanisms listed above regarding the affective eye contact effect. It is possible that all these factors—low-level visual cues of direct gaze, receiving others’ attention, self-referential processing, and interaction—contribute to the positive affective reactions elicited by eye contact. It is also possible that the contribution of these factors vary depending on the way affective reactions are probed in experiments. For future studies, an important aim would be to investigate the specific contribution of these factors on different measures indexing affective reactions.

## Affective Eye Contact Investigated with Images Versus Live Faces

One more important issue related to the suggested mechanisms behind the affective eye contact effects deserves attention. If these effects reflect the influence of receiving others’ attention, interaction, self-referential processing, or some combination of these, how have affective effects also been observed in studies where participants have been shown images of faces, i.e., faces which do not attend to or interact with the observer? As suggested previously, one possibility is that “the belief of being watched” may be an intrinsic property of direct gaze, possibly based on both human evolution and overlearning during early life, and that it is embedded in the perception of direct gaze (Conty et al., 2016). This would explain why direct gaze in pictorial stimuli is also capable of eliciting automatic positive affective responses. However, if so, the next problem is how to explain the findings of some studies that revealed that, while a live individual’s gaze direction influenced these reactions, this was not observed when the same participants were shown the same facial stimuli as images (e.g., Hietanen et al., 2008; Pönkänen et al., 2011b). Notably, this difference is unlikely to relate to some low-level differences in the stimuli used in these studies. For example, both in studies reporting (Lawson, 2015; Chen et al., 2017a) and not reporting (Hietanen et al., 2008; Pönkänen et al., 2011b) affective effects by gaze direction, the stimuli were static images of faces without any dynamic gaze shifts. Additionally, even when the stimuli were video clips in which the model appears very similar (e.g., with occasional blinks) as compared to when shown live through a liquid crystal window, direct gaze does not result in enhanced autonomic responses (Lyyra et al., 2018), unlike that observed in studies that used a live model individual (Hietanen et al., 2008; Helminen et al., 2011; Pönkänen et al., 2011b; Myllyneva and Hietanen, 2015). Moreover, as cited above, the results of Myllyneva and Hietanen (2015, Experiment 1) showing the effect of a live individual’s gaze direction on affective arousal responses when participants believed that the model was able to see them, but not when they believed that the model could not see them, speak against the possibility that low-level visual differences in live versus pictorial stimuli could explain these differences in the results.

One possibility may be related to the nature of responses measured in different studies. The studies reporting no effects of gaze direction with pictures (while observing the effects with live faces; Hietanen et al., 2008; Pönkänen et al., 2011b) measured physiological responses indexing autonomic arousal and the activation of the affective-motivational brain systems, while the studies showing an effect with face pictures relied on behavioral measures sensitive to the cognitive-affective associations between gaze direction and affective information (i.e., Lawson, 2015; Chen et al., 2017a). Perhaps, contextual information about perceiving (just) a picture, regulates the physiological response systems through a top–down process, which inhibits these responses when there is no actual possibility or need to prepare the system for interaction (cf., Myllyneva and Hietanen, 2016).

Another possibility is that these discordant findings are related to attention allocation toward stimuli and cognitive load during stimulus presentation. In studies where pictorial-gaze stimuli have not evoked affective (physiological) responses, participants have been passive observers without a cognitive task (Hietanen et al., 2008; Pönkänen et al., 2011b). Thus, attentional resources could have been directed, not only to the gaze stimuli, but also to the contextual situation where one is facing a computer monitor and is being presented with pictures. Instead, in studies where pictorial-gaze stimuli were observed to have affective effects (i.e., using the affective priming paradigm and implicit association test; Lawson, 2015; Chen et al., 2017a), participants’ attention was directed to a primary cognitive task—affective categorization of the stimuli. Moreover, in the affective priming paradigm (Chen et al., 2017a), participants were even instructed to ignore the gaze stimuli, that is, the primes. In fact, the possibility that these discrepant findings are related to attention and cognitive load during stimulus presentation was directly tested in a study by Conty et al. (2010). They reasoned that direct gaze might evoke amygdala-mediated autonomic arousal response when face stimuli are presented secondary to a main task. To this end, they presented pictorial direct-gaze, averted-gaze, and closed-eyes stimuli concomitantly with a demanding word-spelling task or a simple letter decision task. The results showed greater SCRs to direct gaze compared to averted gaze and closed eyes in the context of the demanding task, but no effect of gaze direction was observed in the context of the simple task. Conty and colleagues interpreted their results referring to the fast-track modulator model proposed by Senju and Johnson (2009) and suggested that, without cognitive load, the arousal response mediated by the subcortical route is inhibited by cortical top–down control.

## Conclusion and Future Directions

I started this review by emphasizing the role of gaze perception in allowing a perceiver to infer the direction of another individual’s attention. Indeed, substantial literature has shown the effects of another individual’s gaze direction on the perceiver’s own attention; another individual’s direct gaze attracts a perceiver’s visual attention and gaze toward the other’s eyes. The attentional effects and prioritized processing of direct gaze have also been central in recent models attempting to describe the various effects of direct gaze and eye contact on cognitive processing (Senju and Johnson, 2009; Conty et al., 2016). The present review shows that there is accumulating evidence that eye contact automatically activates affective systems. Thus, it is likely that the affective processes and reactions also play a role in the various “eye contact effects” (cf., Senju and Johnson, 2009) and “watching eyes effects” (cf., Conty et al., 2016) described previously. However, the initiation of affective processes elicited by eye contact were not explicitly described in these models. For example, Conty et al. (2016) postulated that eye contact initiates, via self-directed attention, a self-referential mode of information processing, i.e., a heightened processing of stimuli in relation with the self, and that this leads to the enhancement of self-awareness, memory effects, activation of pro-social behavior, and positive appraisals of others. Now, as eye contact seems to trigger positively valenced affective processing and bodily responses, it is possible that these reactions contribute to the advantageous effect of direct gaze on memory, pro-social behavior, and evaluation of others (for reviews of these effects, see Senju and Johnson, 2009; Conty et al., 2016). The advantageous effects of positive affect, in general, on memory, pro-social behavior, and individual perception are well-documented in the literature (Forgas and Bower, 1987; George, 1991; Ashby et al., 2002).

This research field is abundant with interesting questions waiting to be investigated. Given that research has started to reveal automatic positive affective reactions to eye contact, these findings can pave the way for a broader investigation of these effects in various types of social encounters. Positive affect is known to positively influence performance on a variety of cognitive tasks (Isen, 1999), possibly via increased brain dopamine levels (Ashby et al., 1999). Although longer periods of eye contact may be disruptive for cognitive performance and may lead to gaze aversion (presumably to decrease cognitive load, e.g., Doherty-Sneddon and Phelps, 2005), shorter periods of eye contact could, indeed, trigger positive affective reactions, thus leading to improved cognitive performance and facilitation of social interaction. A particularly interesting issue relates to the possible effects of eye contact on therapeutic change, via positive affective reactions. In the field of psychotherapy, positive affect has been suggested to play a role as a generator of therapeutic change by facilitating cognitive flexibility (Fitzpatrick and Stalikas, 2008).

More specific issues deserving future research relate, for example, to the role of physical presence in the affective eye contact effect. If positive affective influences reflect the understanding of being attended by others or the possibility for reciprocal interaction, what role does physical presence play in eliciting these affective reactions? Will eye contact elicit positive affective reactions when seeing another via a telecommunication application as well? So far, everyday experiences suggest that this might not be the case, but this may be due to the typical technical limitations (e.g., location of the camera in relation to the screen and time-delay in transmitting the video signal). Another, highly interesting field relates to human interaction with robots. Presently, technology in the field of robotics is developing fast and social robots are starting to appear. Just within a few years, we may be interacting not only with fellow humans but also with robots in our homes, workplaces, and in places offering various services. How do we react affectively to robots, and is eye contact with a robot capable of eliciting similar kinds of positive affective reactions as eye contact with another human does? If it turns out that eye contact with robots also generates positive affective reactions in humans, this could have huge potential in terms of using robots to increase people’s well-being and to alleviate negative states of feelings, for example, in people having difficulties in forming and maintaining social relations with others (e.g., due to psychological problems or physical handicaps) or in people suffering from loneliness.

John Heron once wrote: “The most fundamental primary mode of interpersonal encounter is the interaction between two pairs of eyes and what is mediated by this interaction” (Heron, 1970, p. 244). The present review suggests that this encounter not only opens a door for the meeting of minds, but it does it in an inherently positive way.

## Author Contributions

The author confirms being the sole contributor of this work and approved it for publication.

## Conflict of Interest Statement

The author declares that the research was conducted in the absence of any commercial or financial relationships that could be construed as a potential conflict of interest.
